# Assessing Influenza and SARS‐CoV‐2 Coinfection in Brazil: A Comprehensive Study of Patient Outcomes From 2020 to 2023

**DOI:** 10.1002/jmv.70639

**Published:** 2025-10-11

**Authors:** L. T. A. Gaklik, B. M. Carneiro

**Affiliations:** ^1^ Immunology and Virology Laboratory, School of Health Sciences Federal University of Rondonópolis Rondonópolis Mato Grosso Brazil

**Keywords:** coinfection, COVID‐19, emerging diseases, influenza, respiratory diseases, virus

## Abstract

Influenza and SARS‐CoV‐2 are major respiratory pathogens that have impacted global health, sharing similar transmission routes and clinical symptoms. The COVID‐19 pandemic brought attention to coinfection with these viruses, which has been associated with worse clinical outcomes, but the full extent of this impact remains underexplored. As both viruses circulate together during seasonal outbreaks, understanding their coinfection dynamics is crucial for public health response. This retrospective observational study analyzed data from over 30 000 hospitalized patients sourced from the Brazilian Epidemiological Surveillance System (SIVEP‐Gripe). Patients were classified into two groups: influenza mono‐infection and influenza‐SARS‐CoV‐2 coinfection. Descriptive statistics and multivariate logistic regression were performed to evaluate associations with primary (mortality) and secondary (ICU admission) outcomes. Among approximately 3.7 million reported severe acute respiratory syndrome cases, 35 831 were influenza‐infected, with 1763 (4.9%) coinfected with SARS‐CoV‐2. Coinfected patients exhibited nearly double the risk of death (aOR: 1.87, 95% CI: 1.52−2.30) and a significantly higher likelihood of ICU admission (aOR: 1.27, 95% CI: 1.07–1.52), compared to those with influenza alone. Coinfected patients also presented with more severe respiratory symptoms and longer hospital stays. Coinfection with influenza and SARS‐CoV‐2 is associated with significantly worse clinical outcomes, including higher mortality and increased need for intensive care. Early identification and tailored management strategies for coinfected patients are essential to improving patient outcomes, particularly for those with underlying comorbidities.

## 1 Introduction

Viral infections are significant causes of respiratory tract diseases and, throughout history, have had a profound impact on the socioeconomic status and health of populations. For the past 30 years, infectious respiratory diseases have remained among the leading causes of mortality worldwide [1]. Until 2019, the influenza virus, responsible for the flu, was one of the leading causes of mortality from respiratory diseases in Brazil [2].

In 2020, we experienced the COVID‐19 pandemic, a disease caused by the SARS‐CoV‐2 respiratory virus initially identified in China in December 2019 [3]. Although they are phylogenetically distinct viruses, from a clinical point of view, the diseases caused by these two agents are similar. In the early stages of the pandemic, health teams faced challenges in distinguishing between influenza and COVID‐19 based solely on symptoms. Accurate differentiation of the two infections required careful evaluation of their distinct characteristics, while also considering the potential for coinfection with both pathogens [4].

Since the first cases reported in 2019 and the subsequent pandemics, SARS‐CoV‐2 has continued to circulate in different variant forms, with a seasonality similar to that of other respiratory viruses. It is now widely accepted that SARS‐CoV‐2 is considered an endemic virus in the global landscape [5, 6]. In the context of respiratory viruses, it is well established that multiple viruses co‐circulate during certain seasons, often leading to cases of coinfection [7]. Depending on the specific viruses involved, these coinfections can lead to a worsening of patient outcomes when compared to single infections [8, 9, 10]. As SARS‐CoV‐2 and influenza are currently two of the most widely circulating viruses, the likelihood of co‐infection between them is high.

In the context of influenza and SARS‐CoV‐2 co‐infection, a study by Dao et al. [11] revealed that the proportion of coinfection in critically ill COVID‐19 patients was greater than that in patients in the general population. This finding highlighted the importance of screening respiratory pathogens to detect coinfections. On the other hand, a study by Guan et al. [12] demonstrated the opposite findings: coinfection with these two viruses had no effect on overall mortality, and the risk of critical outcomes was lower in patients with co‐infection. Additionally, different associations were found in different regions, which drew attention to the order in which the viruses coinfected patients.

Coinfections also represent an additional challenge during respiratory disease outbreaks, as they can overwhelm health systems, especially during peaks in the incidence of each disease, impacting public policies. Despite increasing reports of coinfections, the literature currently presents contradictory findings regarding the severity and clinical outcomes of co‐infection between influenza and SARS‐CoV‐2, particularly in settings where both viruses are circulating simultaneously. This study, therefore, aims to explicitly compare the clinical outcomes of influenza mono‐infection and influenza‐SARS‐CoV‐2 co‐infection. Additionally, it seeks to describe the epidemiological and clinical characteristics of the patients included in the study, addressing the need for a more consistent understanding of how these coinfections influence disease progression and patient management, with a large cohort of patients that may contribute to clarifying the conflicting results in the literature.

## 2 Materials and Methods

### 2.1 Study Design & Participants

This study is a retrospective observational analysis using the public and anonymized Severe Acute Respiratory Syndrome (SARS) database from the Influenza Epidemiological Surveillance Information System (SIVEP‐Gripe). The study included patients who were registered in the SIVEP‐Gripe database, with a confirmed laboratory diagnosis (molecular or serological) of influenza or SARS‐CoV‐2 infection. Inclusion criteria were: (a) a confirmed positive laboratory result for either influenza or SARS‐CoV‐2 and (b) symptom onset between January 2020 and December 2023. Patients who were only clinically or radiologically diagnosed, those who were not hospitalized, or those with missing data on variables required for determining inclusion or exclusion were excluded from the study.

### 2.2 Data Source

The data for this study were sourced from the Influenza Epidemiological Surveillance Information System (SIVEP‐Gripe, *Sistema de Informação da Vigilância Epidemiológica da Gripe*), a national surveillance database maintained by the Brazilian Ministry of Health. SIVEP‐Gripe serves as the primary instrument for monitoring Severe Acute Respiratory Infections (SARI) throughout Brazil. Notification of all individuals hospitalized with SARI is mandatory for all public and private healthcare facilities, including clinics and hospitals across all complexity levels. This comprehensive and compulsory reporting framework ensures broad national coverage, making SIVEP‐Gripe a robust and representative data source for analyzing SARI trends and outcomes in the country.

Within this system, a SARI case is defined as a patient presenting with at least two of the following symptoms: fever (even if referred), chills, sore throat, headache, cough, runny nose, olfactory or taste disturbances, and at least one severe symptom: dyspnea/respiratory distress, persistent chest pressure, O_2_ saturation less than 95% on room air, or bluish discoloration of the face. For each notified case, the database includes sociodemographic data, comorbidities, clinical signs, risk factors, and the suspected etiological agent.

### 2.3 Covariables

The SARS notification form includes more than 150 data fields covering various patient dimensions. For this study, we focused on the collection of sociodemographic information, comorbidities, symptoms, and risk factors. The information was organized into blocks according to this classification and analyzed according to this division.

Sociodemographic information included variables such as sex, age, ethnicity, pregnancy status, prior influenza vaccination, and antiviral influenza treatment. Age was categorized into five groups: less than 1 year, 1−5 years, 5−16 years, 16−65 years, and older than 65 years. Pregnancy status was classified as yes or no at the time of hospitalization. Influenza vaccination status referred to whether the patient had received the vaccine in the previous vaccination campaign (within the last 12 months). Antiviral treatment was recorded if the patient had received influenza antiviral medications (Oseltamivir or Zanamivir) immediately before or during hospitalization.

The presence of any comorbidity was recorded, with individual categories including postpartum, heart disease, hematologic disease, Down syndrome, liver disease, asthma, diabetes, neurological disorders, chronic pulmonary disease, immunosuppression, kidney disease, and obesity. Symptoms collected included fever, cough, sore throat, dyspnea, respiratory distress, oxygen saturation < 95%, diarrhea, vomiting, abdominal pain, fatigue, anosmia, and ageusia.

Risk factors analyzed included time to hospitalization (the time between symptom onset and hospitalization), length of hospital stay (duration between hospitalization and case closure), time to case closure (discharge/recovery or death), intensive care unit (ICU) admission (whether the patient required ICU care), length of ICU stay (calculated as the time from ICU admission to case closure), type of ventilatory support (whether invasive or noninvasive support was required), and case closure (categorized as death, recovery, or death by other causes).

### 2.4 Study Outcomes

The study assessed two primary clinical endpoints. The primary outcome was in‐hospital mortality, defined as a case closure record indicating death directly attributed to the acute respiratory infection during the hospitalization period. The secondary outcome was the need for ICU admission, a binary variable indicating whether a patient required ICU care at any point during their hospitalization.

### 2.5 Data Processing and Analysis

Data were extracted from annual raw files that recorded all SARS notifications during the study period and are available in the Datasus database (https://opendatasus.saude.gov.br/). Owing to the large volume of data, the reading and preliminary analysis of the data were carried out through a script developed in Python, using the *pandas* module for sorting and organizing the information.

The data were initially filtered according to previously established inclusion criteria and were classified as monoinfection or coinfection. The selected variables were subsequently summarized.

For the identification of duplicate data, two complementary approaches were employed. The first utilized the duplicated method from the *pandas* library, which compares data rows sequentially to identify entries that are identical across all columns. The second approach applied a hashing procedure using the SHA‐256 algorithm via Python's *hashlib* library. Each record was encoded and processed with SHA‐256 to generate a unique 64‐character hexadecimal hash. Identical records produced identical hashes, while even minimal differences in the data resulted in distinct outputs. Both methods were executed to ensure comprehensive detection of duplicate entries.

Given that most of our data consists of binary categories, we assessed outliers only for the continuous variables. To detect outliers, we used the interquartile range (IQR) method based on the median, and values were considered acceptable if they were within 1.5 times the IQR above and below the quartiles (25th and 75th). Additionally, for the variables based on dates (time to hospitalization, length of hospital stay, and ICU length of stay), we checked for impossible values (e.g., outside the study period) and potential typographical errors. If any errors were detected in any variable, the corresponding entry was excluded from median calculations and univariate analysis for that category.

We chose not to exclude any entries after applying the inclusion and exclusion criteria. To address the inconsistencies and missing data in the SIVEP database, missing or inconsistent values for binary variables were classified as “Unknown.” For continuous variables, missing or inconsistent values were handled as described above. Only for the multivariate analysis were rows with incomplete data for any of the variables included in the analysis excluded.

### 2.6 Statistical Analysis

To assess the impact of coinfection with influenza and SARS‐CoV‐2, patients were divided into two groups: monoinfected and coinfected. Initially, a descriptive analysis of the data, including absolute (n) and relative (%) frequencies of categorical variables and means, medians, and IQR of continuous variables, was performed. The distribution of the data was verified via the Kolmogorov‒Smirnov test. The Mann‒Whitney test was used to compare continuous variables, whereas Pearson's chi‐square test was used to compare categorical variables between patients diagnosed with simple influenza virus infection and patients coinfected with SARS‐CoV‐2. For all analyses, the level of statistical significance used was 5%. The analyses were conducted via the Python *scipy* library.

To assess the impact of the variables on the primary (death) and secondary (ICU admission) outcomes, multivariate logistic regression was performed. Variables that had a significant result (*p* < 0.05) in the univariate analysis, had data completeness greater than 70%, and had clinical relevance to the study objective were included in both models. The variables were included in steps and added incrementally, analyzing the impact on the AIC (Akaike information criterion) value.

The data from the multivariate analysis were summarized and used to assemble forest graphs, which represent the different impacts of the variables included in the model on the primary and secondary outcomes of the research. The representations were created via the *forestplot* and *matplotlib* modules of the Python language.

### 2.7 Ethical Considerations

This is a publicly available database that has been provided in an anonymous form, with no possibility of individual identification. Therefore, in accordance with national legislation, registration of this project with the Research Ethics Committee is not required.

## 3 Results

In this study, we analyzed the effect of coinfection between the influenza virus and SARS‐CoV‐2 based on public data made available by the Influenza Epidemiological Surveillance System (SIVEP‐Gripe). The analysis revealed that coinfection with influenza and SARS‐CoV‐2 significantly increased the risk of mortality and ICU admission compared to influenza monoinfection. During the period analyzed, approximately 3.7 million notifications of SARS were registered. Among these notifications, the influenza virus (FLU) was identified in 34 566 cases (< 1%), being the exclusive etiological agent in more than 91% of these cases (32 803/35 831). In the context of coinfections, SARS‐CoV‐2 was the most common combination, occurring in 1763 notified patients (58.2%), followed by respiratory syncytial virus (19.2%, 582/3028) and human rhinovirus (3.7%, 111/3028).

In terms of sociodemographic characteristics (Table 1), most individuals in the coinfected group were male, whereas most of the monoinfected individuals were women, indicating a significant variation in the patient profile between the groups. With respect to age, the most frequent category in our data set was individuals older than 65 years, representing 35.2% of the cases. However, a difference was observed in relation to coinfection status: most coinfected patients were aged 16−65 years, whereas single infections were more prevalent among individuals over 65 years of age.

**Table 1:** Sociodemographic and health‐related characteristics of patients hospitalized with influenza virus (FLU) monoinfection compared with those of patients coinfected with FLU and SARS‐CoV‐2, Brazil, from January 2020 to December 2023.

Variables	FLU (*n* = 32 803)	FLU+SARS‐CoV‐2 (*n* = 1763)	OR (raw)	*p* value
Sex	—	—	—	0.000
Male	15 493 (47.2)	948 (53.8)	—	—
Female	17 307 (52.8)	815 (46.2)	0.77	
Age (years)	—	—	—	0.000
< 1	2574 (7.8)	92 (5.2)	—	—
1–5	3992 (12.2)	124 (7.0)	0.87	—
5–16	4764 (14.5)	113 (6.4)	0.66	—
16–65	10 021 (30.5)	731 (41.5)	2.04	—
> 65	11 452 (34.9)	703 (39.9)	1.72	—
Race/ethnicity	—	—	—	0.280
White	14 524 (44.3)	742 (42.1)	—	—
Black	1164 (3.5)	64 (3.6)	1.08	—
Asian	249 (0.8)	20 (1.1)	1.57	—
Mixed‐race	10 994 (33.5)	603 (34.2)	1.07	—
Indigenous	123 (0.4)	6 (0.3)	0.95	—
Unknown	5749 (17.5)	328 (18.6)	1.12	—
Pregnant	—	—	—	0.260
Yes	850 (7.6)	35 (5.9)	0.78	
No	9287 (82.8)	492 (83.4)	—	
Unknown	1082 (9.6)	63 (10.7)	1.10	
Influenza vaccination				0.437
Yes	3332 (10.2)	166 (9.4)	0.95	
No	7889 (24.0)	413 (23.4)	—	
Unknown	21 582 (65.8)	1184 (67.2)	1.05	
Influenza antiviral treatment				0.000
Yes	8358 (25.5)	316 (17.9)	0.57	
No	16 812 (51.3)	973 (55.2)	—	
Unknown	7633 (23.3)	474 (26.9)	1.07	

*Note:* Categorical variables are presented as *n* (%). *Pearson's chi‐square test; ^#^Mann‒Whitney test.

Abbreviations: FLU, influenza; OR, odds ratio.

Regarding influenza vaccination in the previous year, only approximately 10% of hospitalized patients in both groups had been vaccinated, with no significant variation between the groups. Notably, however, there was a high rate of incomplete data (> 60%) for this variable. Also, approximately one quarter of the patients who were monoinfected with influenza used antivirals during hospitalization, while among the coinfected patients, this percentage was significantly lower.

In terms of race, more than 40% of the patients in both groups declared themselves to be white, with no statistically significant differences between the groups. Among women, less than 10% of the cases in both groups involved pregnant women, with similar frequencies between monoinfected and coinfected individuals.

Between the patients included in the study, the most common symptoms were cough, fever, dyspnea, and respiratory distress, all of which were observed in more than 50% patients in both groups. A significant difference was observed in the presence of some symptoms between the groups. Fever, cough, and vomiting were more frequently observed in individuals infected with only the influenza virus. In contrast, more severe symptoms, such as dyspnea, respiratory distress, and low oxygen saturation, were more prevalent in coinfected patients, suggesting a possibly greater severity of the disease in this group (Supporting Information S1: Table 1).

The greatest difference between the groups was observed in the symptoms of anosmia and ageusia, which are characteristic of SARS‐CoV‐2 infection. The frequency of these symptoms in coinfected patients was more than twice as high as that in the monoinfected group, which suggests that coinfection can result in a sum of the typical symptoms of each infection (Supporting Information S1: Table 1).

With respect to risk factors, at least half of the patients in both groups had at least one factor, and this frequency was greater among coinfected patients. Among the most observed factors were heart disease, which was present in almost a quarter of the patients in both groups, and diabetes, which was observed on average in 15% of the individuals included in this study. Notably, among the risk factors significantly different between the groups, only asthma was more common in the simple infection group. In contrast, conditions such as heart disease, diabetes, immunosuppression, and obesity were more frequently observed among coinfected patients (Supporting Information S1: Table 2).

With respect to the variables associated with the main morbidity and mortality factors in this study, a statistically significant difference was observed in most of the variables compared. The median interval between the onset of the first symptoms and hospitalization was only 3 days for coinfected patients, whereas for monoinfected patients, this interval was almost 50% longer (5 days, *p* = 0.000). The length of hospitalization, measured as the difference between the day of admission and the outcome (cure or death), was also greater in coinfected patients (Table 2).

**Table 2:** Morbidity factors and clinical outcomes of patients hospitalized with influenza virus (FLU) monoinfection compared with patients coinfected with FLU and SARS‐CoV‐2, Brazil, January 2020 to December 2023.

	FLU (*n* = 32 803)	FLU+SARS‐CoV‐2 (*n* =1763)	OR (raw)	*p* value
Time to hospitalization (days)	5 (3.0–10.0)	3 (1.0–5.0)		0.000^#^
Length of hospital stay (days)	6 (4.0–11.0)	7 (3.0–15.0)		0.000^#^
ICU admission				0.000
Yes	9347 (28.5)	578 (32.8)	1.25	
No	20 655 (63.0)	1021 (57.9)	—	
Unknown	2801 (8.5)	164 (9.3)	1.18	
ICU length of stay (days)	8 (4.0–15.0)	10 (5.0–21.0)		0.204
Ventilatory support				0.000
Invasive	3862 (11.8)	293 (16.6)	1.70	
Noninvasive	14 217 (43.3)	779 (44.2)	1.23	
None	10 625 (32.4)	474 (26.9)	—	
Unknown	4099 (12.5)	217 (12.3)	1.19	
Outcome				0.000
Recovery	25 864 (78.8)	1215 (68.9)	—	
Death	3787 (11.5)	394 (22.3)	2.21	
Death (other causes)	372 (1.1)	30 (1.7)	1.72	
Unknown	2780 (8.5)	124 (7.0)	0.95	

*Note:* Continuous variables are presented as medians (interquartile ranges), and categorical variables are presented as *n* (%). *Pearson's chi‐square test; ^#^Mann‒Whitney test.

Abbreviations: ICU, intensive care unit; OR, odds ratio.

In addition, the need for admission to ICUs and ventilatory support was more common among coinfected patients, and the greater demand for invasive ventilatory support in this group was especially noteworthy. Finally, the death rate was almost double that of coinfected patients, indicating greater disease severity in these individuals (Table 2).

**Figure 1:**
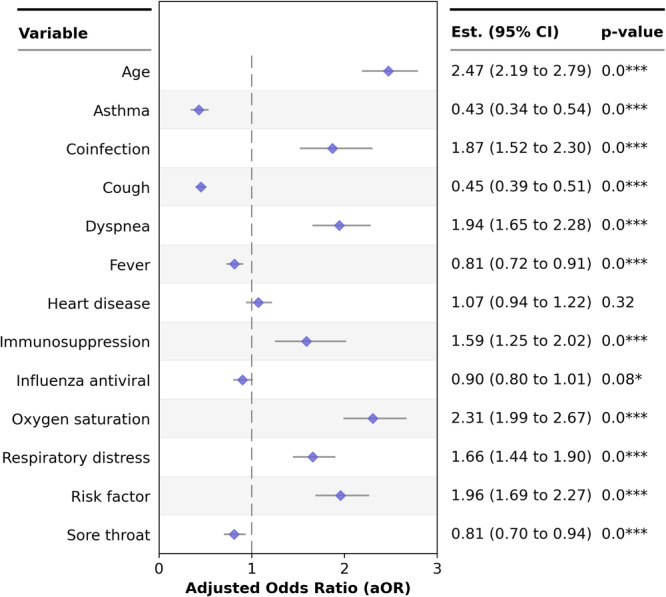
Multivariate logistic regression analysis using patient death as the primary outcome.

To assess the impact of additional variables on the outcome of death, a multivariate logistic regression analysis was performed. The results revealed that coinfected patients were almost twice as likely to die as patients with isolated influenza virus infection were. This analysis also revealed that other variables, such as the presence of risk factors, age over 65 years, dyspnea, and O₂ saturation < 95%, also increased the risk of death by nearly two times or more for coinfected patients (Figure 1).

**Figure 2:**
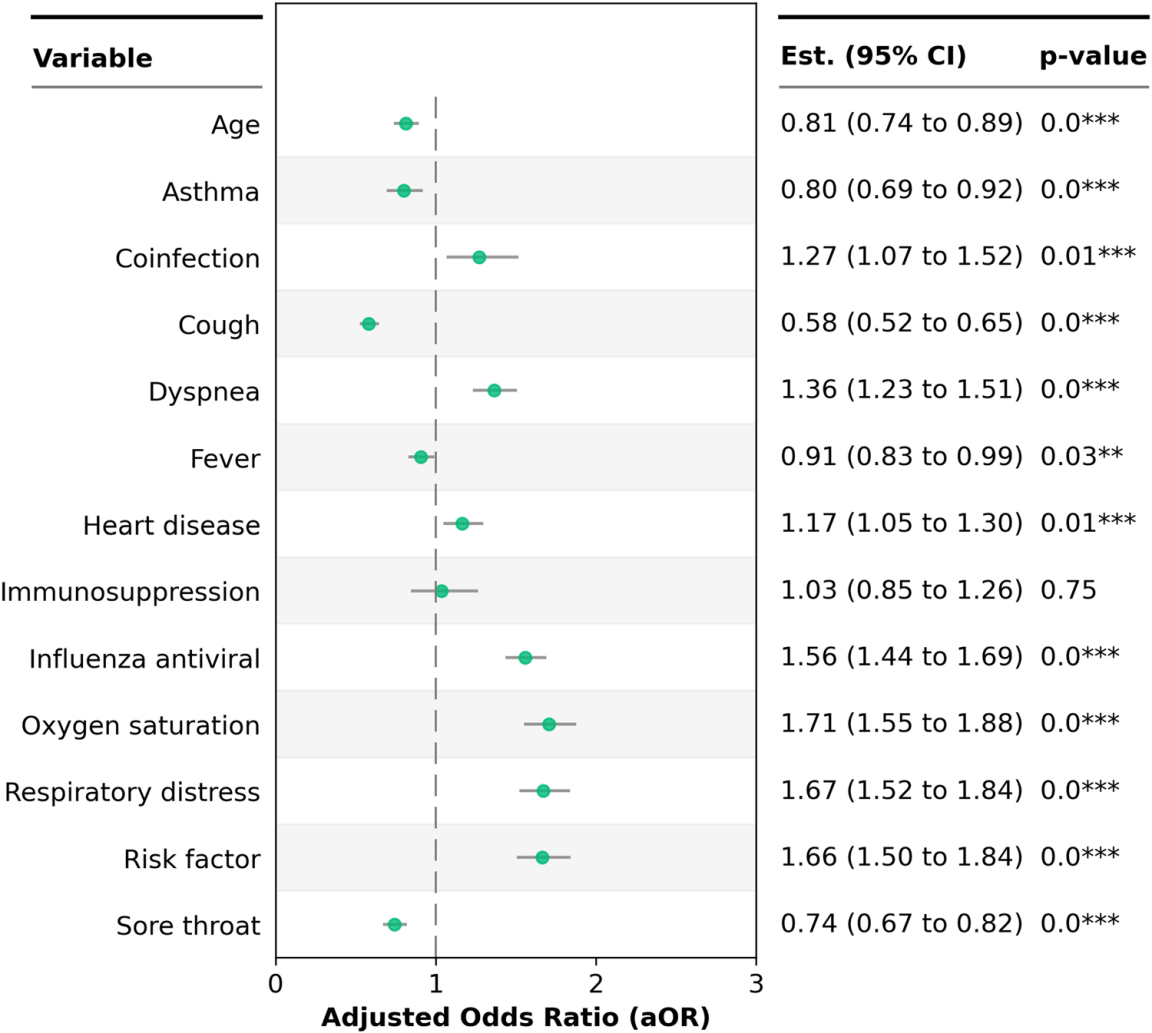
Multivariate logistic regression analysis using patient admission to the ICU as the secondary outcome.

In the same model, the analysis of the secondary outcome—the need for admission to the ICU—indicated that coinfected patients were almost 20% more likely to be admitted to the ICU than those infected with only the influenza virus. In addition to coinfection, other variables also increased the likelihood of ICU admission, including the presence of risk factors, respiratory distress, and O₂ saturation < 95% (Figure 2).

The results of this study clearly demonstrated that hospitalized patients coinfected with influenza and SARS‐CoV‐2 viruses had greater clinical severity, a higher incidence of associated risk factors, and worse clinical outcomes than those monoinfected with influenza. These findings suggest a synergistic interaction between the viruses studied, contributing to the deterioration of the clinical picture and increasing the risk of ICU admission and mortality.

## 4 Discussion

In this study, we analyzed the clinical‐epidemiological profile and outcomes of hospitalized patients with simple influenza virus infection compared with those of patients coinfected with influenza and SARS‐CoV‐2. Coinfection was clearly associated with a significant increase in the severity of cases, corroborating previous findings reported in the literature [13, 14, 15, 16]. Specifically, coinfected patients had a greater demand for invasive ventilatory support, prolonged hospitalizations, and worse clinical outcomes than noninfected patients did, confirming a possible synergistic interaction between the viruses.

An important observation of this study was the shorter interval between the onset of symptoms and hospitalization of the coinfected patients. This rapid clinical progression suggests that coinfection induces a more intense and rapid inflammatory response, as demonstrated by Kinoshita et al. [17], who reported a significant increase in serum IL‐6 levels in patients coinfected with influenza A and SARS‐CoV‐2. This elevation of inflammatory cytokines may explain the rapid clinical deterioration and greater severity of respiratory symptoms observed in this group.

With respect to the immunological mechanisms involved, Kim et al. [15] reported that coinfection promotes severe lymphopenia, significantly impairing the adaptive immune response, especially the CD4^+^ T‐cell response and the production of neutralizing antibodies. These immunological factors seem to contribute directly to the severity of coinfection, hindering the clinical recovery of patients and increasing the risk of mortality, corroborating the findings of this study.

A comparison of the symptoms observed in this study with those reported in other investigations revealed that specific clinical manifestations in influenza‐infected patients, such as anosmia and ageusia, are highly suggestive of coinfection, especially due to the characteristic involvement of SARS‐CoV‐2. Gregianini et al. [18] reported that initial symptoms of coinfection can overlap with those of monoinfections, making immediate differential diagnosis difficult and increasing the importance of simultaneous testing and rigorous epidemiological surveillance.

Despite the relatively low prevalence of coinfections described in several studies [19, 21], the consensus is that coinfections significantly increase the risk of severe clinical outcomes. Yan et al. [16], through meta‐analysis, demonstrated that coinfected patients are more than twice as likely to require invasive mechanical ventilation and admission to ICU than monoinfected patients are, results that are fully compatible with the conclusions obtained in this study.

In addition, observations in animal models and systematic reviews suggest that influenza and SARS‐CoV‐2 viruses can exert independent pathological effects, even when coinfecting the same organism. Kinoshita et al. [17] reported that although both viruses can replicate simultaneously in different lung areas without direct interference, coinfection exacerbates lung inflammation and prolongs the pathological process, contributing to more extensive tissue damage and slower clinical recovery.

Additional studies noted that the severity of coinfection also depends on age and pre‐existing comorbidities [20, 21]. In our study, the increased prevalence of comorbidities, such as diabetes, heart disease, obesity, and immunosuppression, among coinfected patients was notable, reinforcing the idea that these conditions play crucial roles in the susceptibility to and clinical severity of coinfection. These findings are especially relevant for clinical management, indicating that patients with these conditions require more aggressive monitoring and interventions early in the course of the disease.

Although patients older than 65 years demonstrated significantly higher adjusted odds of death, they had lower odds of ICU admission. This finding may reflect clinical decision‐making processes where elderly patients, often with multiple comorbidities or frailty, are less likely to receive intensive care interventions due to considerations of prognosis, patient preferences, or resource allocation [22, 23, 24]. Consequently, a proportion of deaths in this age group may occur outside of the ICU setting.

The variability in clinical severity associated with the presence of comorbidities was also already reported [19], who highlighted a greater severity of respiratory and systemic symptoms in coinfected patients with risk factors such as advanced age and obesity. Although their study revealed that coinfected patients were generally younger and had fewer severe comorbidities, the importance of monitoring vulnerable subgroups with greater care remains evident.

Taken together, our results highlight the clinical significance of coinfections in hospitalized patients. The implementation of more comprehensive and sensitive diagnostic tests for viral and bacterial coinfections is urgently needed, as our findings demonstrate that patients coinfected with influenza and SARS‐CoV‐2 experience worse clinical outcomes compared to those infected with a single virus. Furthermore, these results support the reinforcement and targeted implementation of vaccination campaigns against both influenza and SARS‐CoV‐2, particularly within critical seasonal windows to maximize protection. Additionally, our study underscores the importance of early identification and risk stratification of coinfected patients, which could enable healthcare providers to prioritize intensive monitoring and tailored therapeutic interventions, ultimately improving patient prognosis and resource allocation in hospital settings.

A major limitation of this study was the considerable proportion of incomplete or missing data in the notification system, which is a frequent issue in retrospective studies using secondary data sources [25, 26]. Additionally, molecular testing availability fluctuated throughout the period from 2020 to 2023, potentially impacting case identification. It is also likely that influenza case numbers were underestimated, given that many laboratories prioritized testing for SARS‐CoV‐2, especially at the beginning of the pandemic [27, 28]. Moreover, influenza testing varied widely across Brazilian′s regions, which limited our ability and led to the decision not to conduct a geographic stratification analysis [29]. Another important limitation relates to the absence of information on our database about circulating viral strains and variants—for instance, SARS‐CoV‐2 variants such as Delta, Omicron, and their sublineages, as well as influenza A subtypes (H1N1, H3N2) and influenza B lineages—which could have influenced disease severity and patient outcomes. Key clinical variables such as vaccination coverage, antiviral or other treatment regimens, and the burden on healthcare resources were not comprehensively available and therefore could not be adjusted for in the analyses. Collectively, these factors may limit the generalizability of our findings and highlight the need for future studies to implement rigorous and standardized protocols for data collection and reporting, enabling more robust and representative analyses.

This study reinforces the perception that coinfection with influenza and SARS‐CoV‐2 in hospitalized patients is associated with greater clinical severity and a significant increase in hospital mortality. Strategies aimed at the early diagnosis of coinfections, the strengthening of vaccination campaigns, and the appropriate management of comorbidities are essential to mitigate the clinical and epidemiological impact of these concomitant viral infections.

## Author Contributions

B.M.C. conceptualized the study, developed the scripts, and conducted the final manuscript review. L.T.A.G. performed the data analysis and wrote the manuscript. The Article Processing Charge for the publication of this research was funded by the Coordenação de Aperfeiçoamento de Pessoal de Nível Superior ‐ Brasil (CAPES) (ROR identifier: 00x0ma614).

## Conflicts of Interest

The authors declare no conflicts of interest.

## Supporting information

Supplementary material.

## Data Availability

The data that support the findings of this study are openly available in Síndrome Respiratória Aguda Grave (SRAG) at https://opendatasus.saude.gov.br/.
