# Supplementary material for: Assessing Influenza and SARS‐CoV‐2 Coinfection in Brazil: A Comprehensive Study of Patient Outcomes From 2020 to 2023
Source: J Med Virol. 2025 Oct 11;97(10):e70639. doi: 10.1002/jmv.70639 (PMC12514659; doi:10.1002/jmv.70639)
Supplement: Supplementary file 1 — Supplementary material. [file JMV-97-e70639-s001.docx]

**Assessing influenza and SARS-CoV-2 coinfection in Brazil: A comprehensive study of patient outcomes from 2020 to 2023.**

Gaklik, L. T. A.**^1^**; Carneiro, B. M.**^1*^**

**Supplementary material**

**Supplementary table 1** – Clinical characteristics of patients hospitalized with influenza virus (FLU) monoinfection compared with those of patients coinfected with FLU and SARS-CoV-2, Brazil, from Jan 2020 to Dec 2023.

|  | **FLU**  **(n=32,803)** | **FLU + SARS-CoV-2**  **(n = 1.763)** | **OR**  **(Raw)** | **p -value** |
| --- | --- | --- | --- | --- |
| **Fever** |  |  |  | 0.000 |
| Yes | 22,434 (68.4) | 1,062 (60.2) | 0.74 |  |
| No | 6,214 (18.9) | 395 (22.4) | - |  |
| Unknown | 4,155 (12.7) | 306 (17.4) | 1.16 |  |
| **Cough** |  |  |  | 0.000 |
| Yes | 26,356 (80.3) | 1,335 (75.7) | 0.80 |  |
| No | 3,627 (11.1) | 231 (13.1) | - |  |
| Unknown | 2,820 (8.6) | 197 (11.2) | 1.10 |  |
| **Sore throat** |  |  |  | 0.003 |
| Yes | 5,847 (17.8) | 319 (18.1) | 1.10 |  |
| No | 16,995 (51.8) | 846 (48.0) | - |  |
| Unknown | 9,961 (30.4) | 598 (33.9) | 1.21 |  |
| **Dyspnea** |  |  |  | 0.000 |
| Yes | 19,600 (59.8) | 1,116 (63.3) | 1.34 |  |
| No | 8,063 (24.6) | 342 (19.4) | - |  |
| Unknown | 5,140 (15.7) | 305 (17.3) | 1.40 |  |
| **Respiratory distress** |  |  |  | 0.007 |
| Yes | 17,097 (52.1) | 932 (52.9) | 1.14 |  |
| No | 8,934 (27.2) | 427 (24.2) | - |  |
| Unknown | 6,772 (20.6) | 404 (22.9) | 1.25 |  |
| **Oxygen saturation < 95%** |  |  |  | 0.000 |
| Yes | 16,544 (50.4) | 960 (54.5) | 1.34 |  |
| No | 9,804 (29.9) | 424 (24.0) | - |  |
| Unknown | 6,455 (19.7) | 379 (21.5) | 1.36 |  |
| **Diarrhea** |  |  |  | 0.000 |
| Yes | 2,521 (7.7) | 161 (9.1) | 1.30 |  |
| No | 19,706 (60.1) | 966 (54.8) | - |  |
| Unknown | 10,576 (32.2) | 636 (36.1) | 1.23 |  |
| **Vomiting** |  |  |  | 0.003 |
| Yes | 3,684 (11.2) | 163 (9.2) | 0.86 |  |
| No | 18,841 (57.4) | 975 (55.3) | - |  |
| Unknown | 10,278 (31.3) | 625 (35.5) | 1.18 |  |
| **Abdominal pain** |  |  |  | 0.005 |
| Yes | 1,810 (5.5) | 93 (5.3) | 0.47 |  |
| No | 18,626 (56.8) | 937 (53.1) | - |  |
| Unknown | 12,367 (37.7) | 733 (41.6) | 0.55 |  |
| **Fatigue** |  |  |  | 0.000 |
| Yes | 5,570 (17.0) | 362 (20.5) | 1.38 |  |
| No | 15,733 (48.0) | 741 (42.0) | - |  |
| Unknown | 11,500 (35.1) | 660 (37.4) | 1.22 |  |
| **Anosmia** |  |  |  | 0.000 |
| Yes | 452 (1.4) | 52 (2.9) | 2.36 |  |
| No | 19,548 (59.6) | 951 (53.9) | - |  |
| Unknown | 12,803 (39.0) | 760 (43.1) | 1.22 |  |
| **Ageusia** |  |  |  | 0.000 |
| Yes | 479 (1.5) | 55 (3.1) | 2.37 |  |
| No | 19,459 (59.3) | 941 (53.4) | - |  |
| Unknown | 12,865 (39.2) | 767 (43.5) | 1.23 |  |

Categorical variables are presented as n (%). *Pearson's chi-square test. OR: odds ratio

**Supplementary table 2** – Main comorbidities among patients hospitalized with influenza virus (FLU) monoinfection compared with those coinfected with FLU and SARS-CoV-2, Brazil, from Jan 2020 to Dec 2023.

|  | **FLU**  **(n=32,803)** | **FLU + SARS-CoV-2**  **(n = 1.763)** | **OR**  **(Raw)** | **p -value** |
| --- | --- | --- | --- | --- |
| **Risk factor (any)** |  |  |  | 0.000 |
| Yes | 17,931 (54.7) | 1,096 (62.2) | 1.36 |  |
| No | 14,872 (45.3) | 667 (37.8) | - |  |
| **Postpartum** |  |  |  | 0.580 |
| Yes | 158 (8.7) | 9 (1.1) | 1.21 |  |
| No | 17,149 (91.3) | 806 (98.9) | - |  |
| **Heart disease** |  |  |  | 0.000 |
| Yes | 7,563 (23.1) | 506 (28.7) | 1.35 |  |
| No | 25,135 (76.6) | 1,248 (70.8) | - |  |
| Unknown | 105 (0.3) | 9 (0.5) | 1.73 |  |
| **Hematologic disease** |  |  |  | 0.151 |
| Yes | 361 (1.1) | 25 (1.4) | 1.30 |  |
| No | 32,284 (98.4) | 1,725 (97.8) | - |  |
| Unknown | 158 (0.5) | 13 (0.7) | 1.54 |  |
| **Down syndrome** |  |  |  | 0.245 |
| Yes | 169 (0.5) | 7 (0.4) | 0.77 |  |
| No | 32,507 (99.1) | 1,745 (99.0) | - |  |
| Unknown | 127 (0.4) | 11 (0.6) | 1.61 |  |
| **Liver disease** |  |  |  | 0.186 |
| Yes | 261 (0.8) | 13 (0.7) | 0.93 |  |
| No | 32,398 (98.8) | 1,737 (98.5) | - |  |
| Unknown | 144 (0.4) | 13 (0.7) | 1.68 |  |
| **Asthma** |  |  |  | 0.000 |
| Yes | 2.636 (8.0) | 101 (5.7) | 0.70 |  |
| No | 30,034 (91.6) | 1,648 (93.5) | - |  |
| Unknown | 133 (0.4) | 14 (0.8) | 1.92 |  |
| **Diabetes** |  |  |  | 0.000 |
| Yes | 4,734 (14.4) | 335 (19.0) | 1.39 |  |
| No | 27,957 (85.2) | 1,420 (80.5) | - |  |
| Unknown | 112 (0.3) | 8 (0.5) | 1.41 |  |
| **Neurological disorders** |  |  |  | 0.124 |
| Yes | 1,678 (5.1) | 87 (4.9) | 0.97 |  |
| No | 30,989 (94.5) | 1,663 (94.3) | - |  |
| Unknown | 136 (0.4) | 13 (0.7) | 1.78 |  |
| **Chronic pulmonary disease** |  |  |  | 0.381 |
| Yes | 2,332 (7.1) | 130 (7.4) | 1.04 |  |
| No | 30,335 (92.5) | 1,622 (92.0) | - |  |
| Unknown | 136 (0.4) | 11 (0.6) | 1.51 |  |
| **Immunosuppression** |  |  |  | 0.000 |
| Yes | 1,022 (3.1) | 116 (6.6) | 2.19 |  |
| No | 31,634 (96.4) | 1,636 (92.8) | - |  |
| Unknown | 147 (0.4) | 11 (0.6) | 1.45 |  |
| **Kidney disease** |  |  |  | 0.050 |
| Yes | 974 (3.0) | 57 (3.2) | 1.10 |  |
| No | 31,681 (96.6) | 1,691 (95.9) | - |  |
| Unknown | 148 (0.5) | 15 (0.9) | 1.90 |  |
| **Obesity** |  |  |  | 0.000 |
| Yes | 1,111 (3.4) | 107 (6.1) | 1.85 |  |
| No | 31,489 (96.0) | 1,641 (93.1) | - |  |
| Unknown | 203 (0.6) | 15 (0.9) | 1.42 |  |

Categorical variables are presented as n (%). *Pearson's chi-square test; OR: odds ratio
